# Use of a knotted guidewire to manage duodenoscope working channel obstruction

**DOI:** 10.1016/j.vgie.2025.01.004

**Published:** 2025-01-17

**Authors:** Jun Kubota, Sakue Masuda, Kazuya Koizumi, Makomo Makazu, Karen Kimura

**Affiliations:** Gastroenterology Medicine Center, Shonan Kamakura General Hospital, Kanagawa, Kanagawa, Japan

Endoscopic working-channel blockage often is caused by the accumulation of foreign objects such as tissue fragments or insturuments.^1^^,^^2^ We report an instance in which an endoscopic scope working-channel occlusion, estimated to cost approximately $700 to $2500 for repair, was successfully avoided. A patient with acute cholecystitis managed with a gallbladder stent experienced a recurrence; therefore, we attempted to replace the gallbladder stent using a TJF-Q290 V endoscope (Olympus, Tokyo, Japan), which is equipped with a 4.2-mm working channel. The existing stent (7F 15-cm pigtail plastic stent) was grasped approximately 5 cm from the end of the tube using grasping forceps during its removal. However, the stent detached from the grasping forceps inside the scope and could not be retrieved (Fig. 1, Video 1, available online at www.videogie.org). The endoscope was removed, and attempts to push out the stent using forceps and cleaning devices were unsuccessful (Fig. 2). A guidewire (VisiGlide2, 0.025-inch; Olympus) was then inserted from the proximal side into the working channel and passed beside the stent and through the distal tip of the endoscope (Fig. 3). Subsequently, some knots were made on the working channel side (Fig. 4); the stent was successfully removed by pulling the guidewire toward the tip of the scope after it was caught by the knot (Figs. 5, 6, and 7). The knot loops were repeated 3 times to ensure sufficient hold and stability. After forming 2 loops, a third was passed through 1 of the previous loops to enhance its 3-dimensional structure. When stent grasping failure occurs, increasing the number of loops may improve the rate of success. To address concerns about potential channel blockage or damage caused by the guidewire, we initially opted for a guidewire comprising soft material. The stent was not cut, and insufficient gripping by the forceps was identified as the cause. This procedure is further explained using a model with a transparent tube (Video 1). When all devices pushed from the working channel side fail to retrieve the stent, pulling with a knotted guidewire, as in this case, can be effective. This technique successfully avoids repair costs.

**Figure 1:**
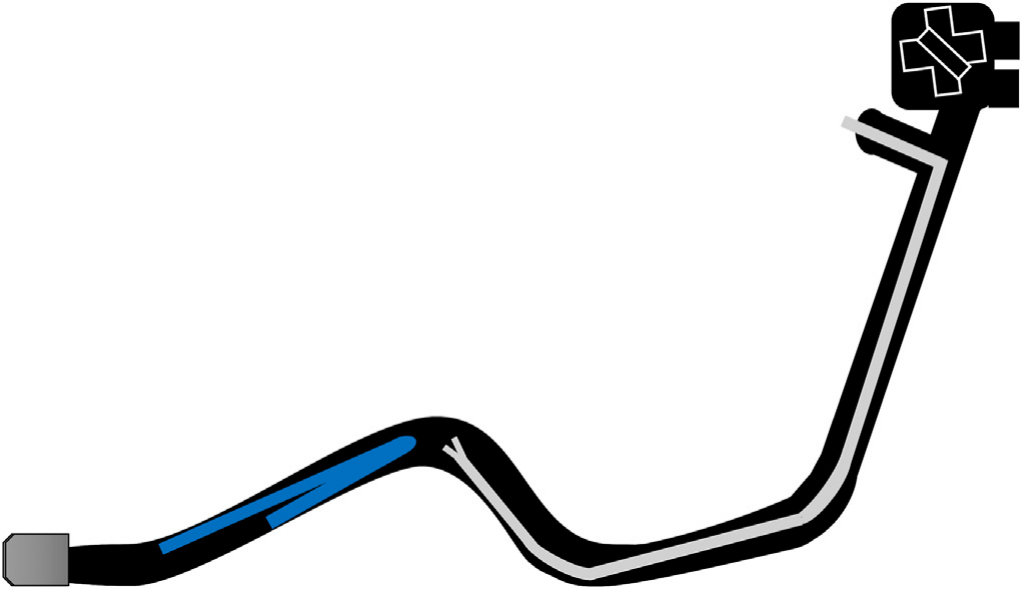
During the removal of the existing stent (7F 15-cm pigtail plastic stent) using grasping forceps, the stent detached from the forceps inside the scope and could not be retrieved.

**Figure 2:**
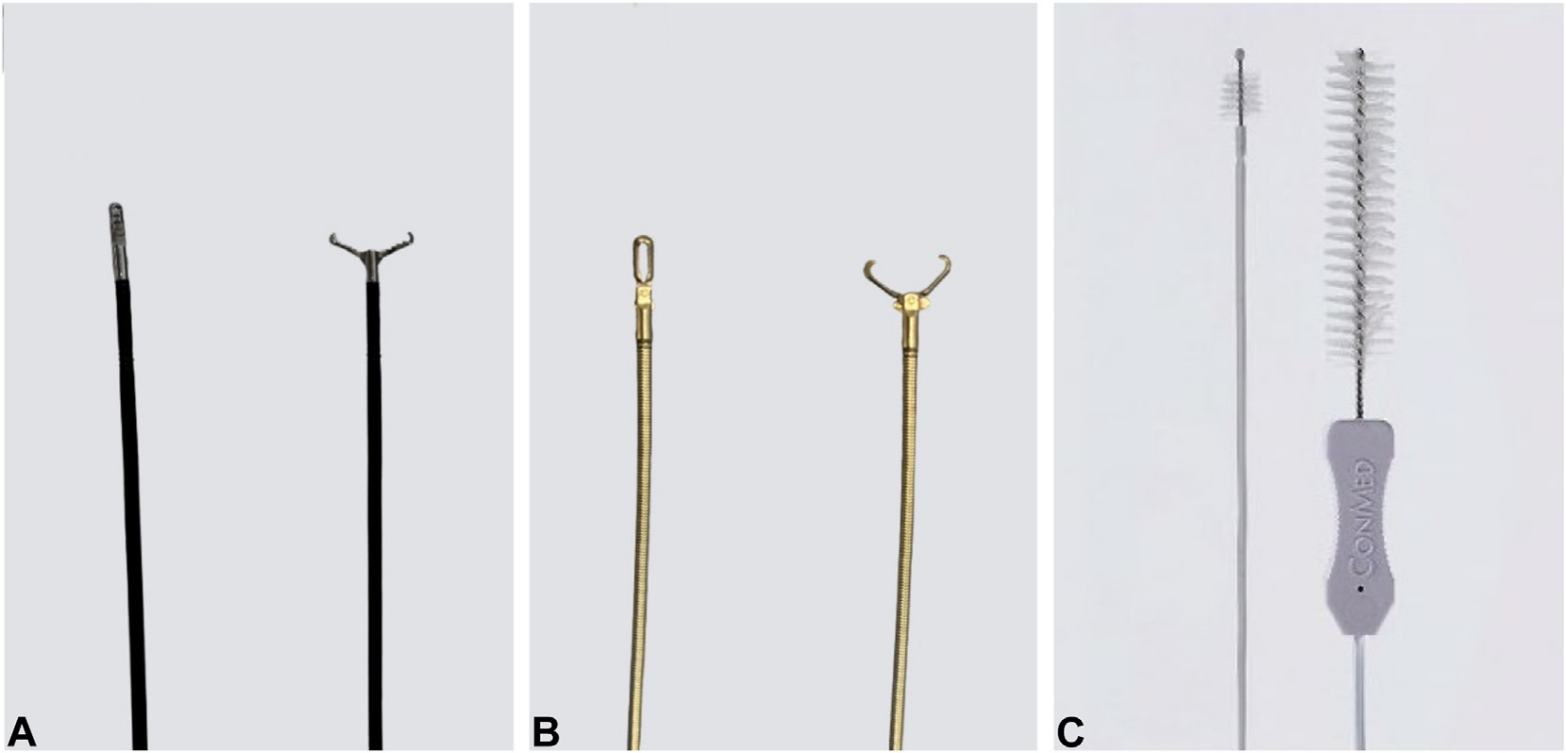
**A,** Grasping forceps. **B,** Removal forceps. **C,** Cleaning device.

**Figure 3:**
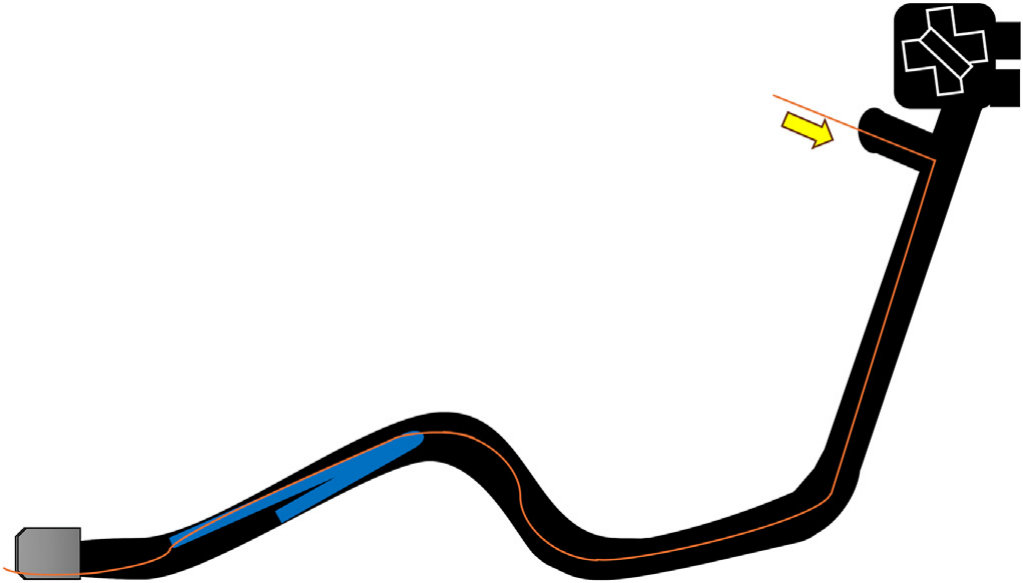
A guidewire (VisiGlide2, 0.0250-inch; Olympus) was inserted from the proximal side into the working channel and passed beside the stent and through the distal tip of the endoscope.

**Figure 4:**
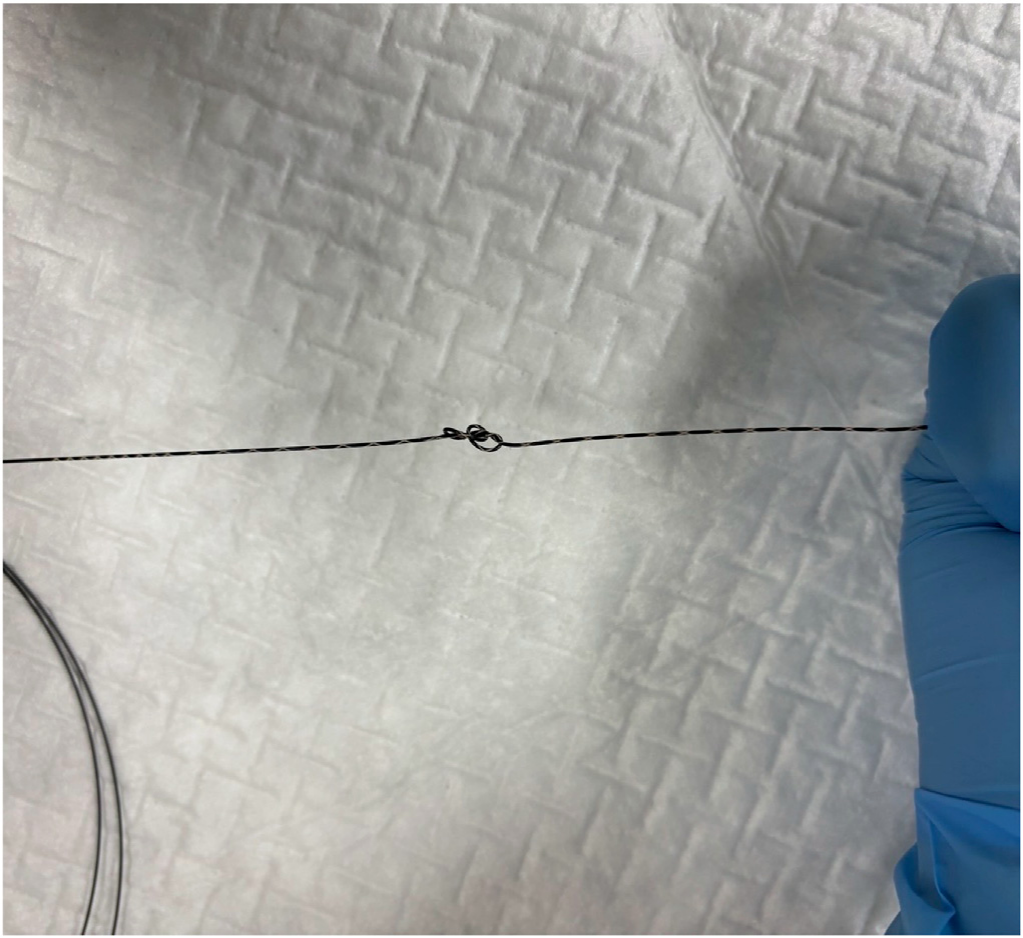
Knots were made on the working channel side.

**Figure 5:**
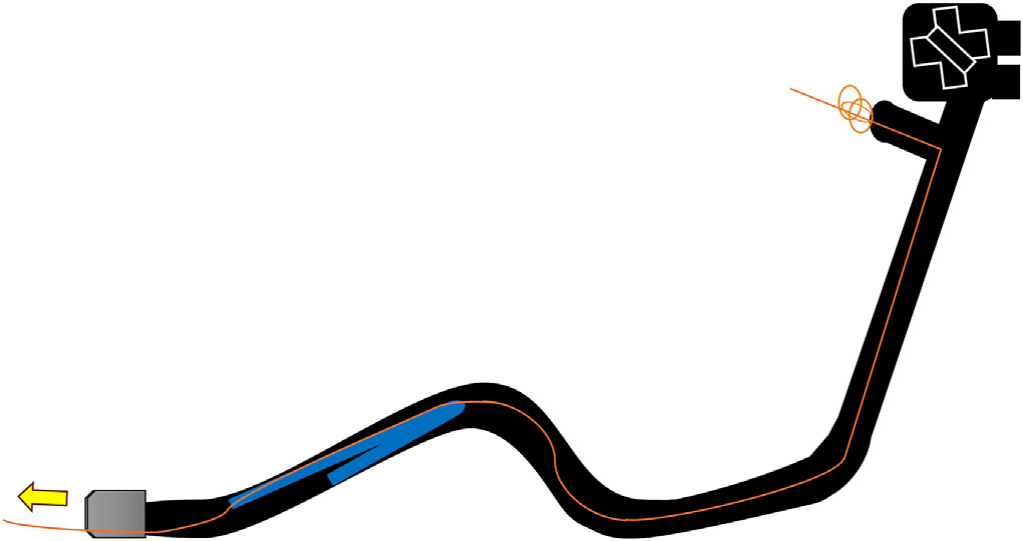
The guidewire was pulled toward the tip of the scope.

**Figure 6:**
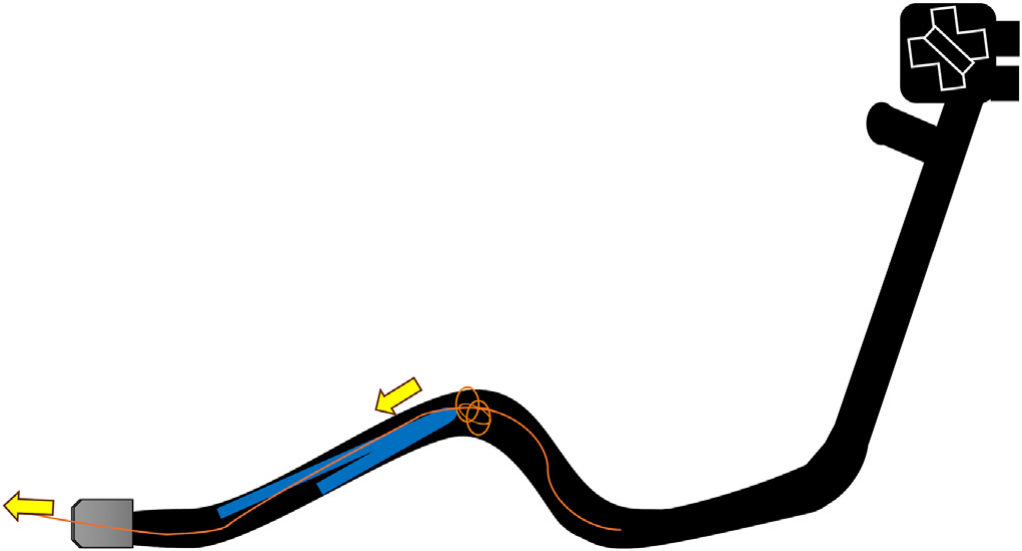
The stent was caught on the knot and pulled up to the tip.

**Figure 7:**
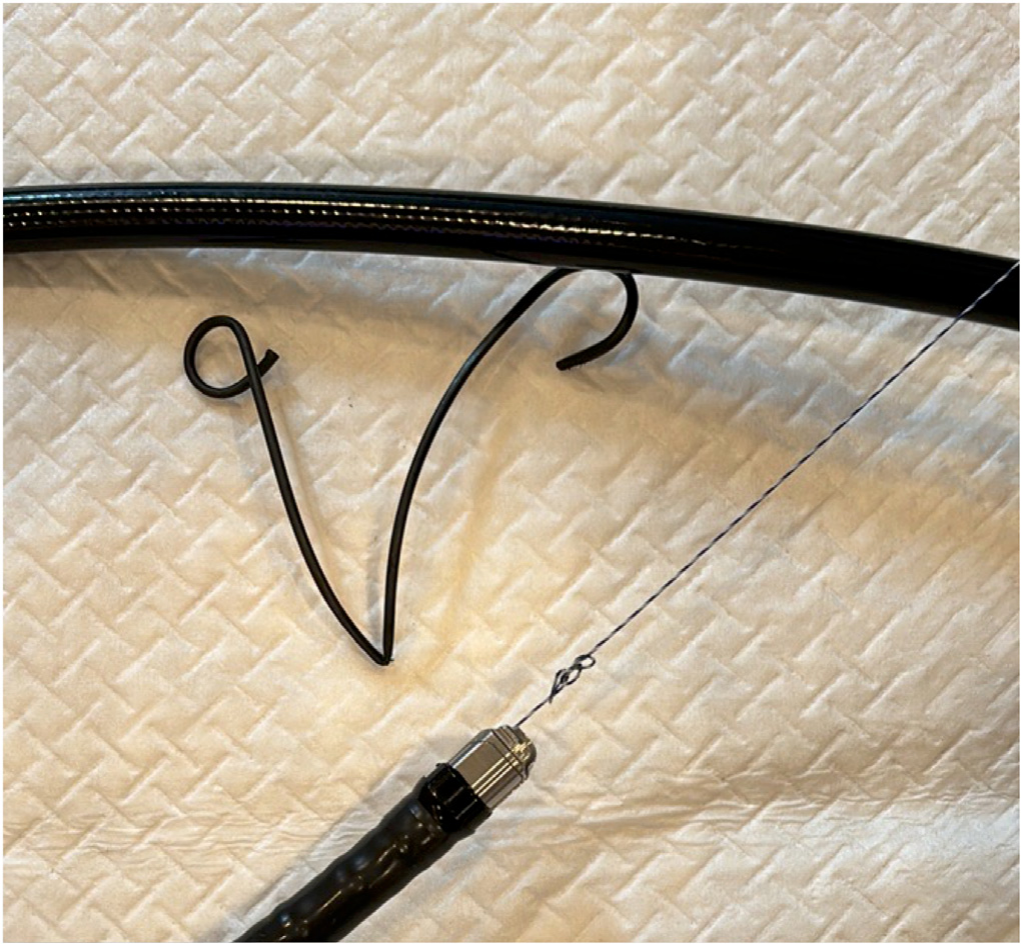
The stent was successfully removed from the scope.

## Patient Consent

The patient in this article has given written informed consent to publication of the case details.

## Disclosures

All authors disclosed no financial relationships.

## Supplementary data

Video 1 Using a guidewire to release the occlusion of the endoscope's working channel caused by a stent.

## References

[1] Lambour A.J., Billmeier S.E., Nau P. (2020). The SAGES Manual of Flexible Endoscopy.

[2] Vitale G.C., Davis B.R., Soper N.J., Scott-Conner C.E.H. (2012). The SAGES manual: Volume 1 Basic Laparoscopy and Endoscopy.

